# The Medical Duty Officer: An Attempt to Mitigate the Ambulance At-Hospital Interval

**DOI:** 10.5811/westjem.2016.7.30266

**Published:** 2016-08-23

**Authors:** Megan H. Halliday, Andrew J. Bouland, Benjamin J. Lawner, Angela C. Comer, Daniel C. Ramos, Mark Fletcher

**Affiliations:** * University of Maryland School of Medicine, Baltimore, Maryland; † University of Maryland School of Medicine, Department of Emergency Medicine, Baltimore Maryland; ‡ Baltimore City Fire Department, Division of EMS, Baltimore, Maryland; § National Study Center for Emergency Medical Systems and Trauma, Baltimore Maryland; ¶ Baltimore City Department of Social Services, Baltimore, Maryland

## Abstract

**Introduction:**

A lack of coordination between emergency medical services (EMS), emergency departments (ED) and systemwide management has contributed to extended ambulance at-hospital times at local EDs. In an effort to improve communication within the local EMS system, the Baltimore City Fire Department (BCFD) placed a medical duty officer (MDO) in the fire communications bureau. It was hypothesized that any real-time intervention suggested by the MDO would be manifested in a decrease in the EMS at-hospital time.

**Methods:**

The MDO was implemented on November 11, 2013. A senior EMS paramedic was assigned to the position and was placed in the fire communication bureau from 9 a.m. to 9 p.m., seven days a week. We defined the pre-intervention period as August 2013 – October 2013 and the post-intervention period as December 2013 – February 2014. We also compared the post-intervention period to the “seasonal match control” one year earlier to adjust for seasonal variation in EMS volume. The MDO was tasked with the prospective management of city EMS resources through intensive monitoring of unit availability and hospital ED traffic. The MDO could suggest alternative transport destinations in the event of ED crowding. We collected and analyzed data from BCFD computer-aided dispatch (CAD) system for the following: ambulance response times, ambulance at-hospital interval, hospital diversion and alert status, and “suppression wait time” (defined as the total time suppression units remained on scene until ambulance arrival). The data analysis used a pre/post intervention design to examine the MDO impact on the BCFD EMS system.

**Results:**

There were a total of 15,567 EMS calls during the pre-intervention period, 13,921 in the post-intervention period and 14,699 in the seasonal match control period one year earlier. The average at-hospital time decreased by 1.35 minutes from pre- to post-intervention periods and 4.53 minutes from the pre- to seasonal match control, representing a statistically significant decrease in this interval. There was also a statistically significant decrease in hospital alert time (approximately 1,700 hour decrease pre- to post-intervention periods) and suppression wait time (less than one minute decrease from pre- to post- and pre- to seasonal match control periods). The decrease in ambulance response time was not statistically significant.

**Conclusion:**

Proactive deployment of a designated MDO was associated with a small, contemporaneous reduction in at-hospital time within an urban EMS jurisdiction. This project emphasized the importance of better communication between EMS systems and area hospitals as well as uniform reporting of variables for future iterations of this and similar projects.

## INTRODUCTION

With a constantly increasing demand on the healthcare system, hospitals and emergency departments (EDs) are faced with increasing numbers of patients each year without a corresponding increase in resources. Hospitals are unable to handle the surges in demand for inpatient beds, which ultimately manifests as ED crowding.^1^ A downstream consequence of ED crowding is the increase in time an ambulance waits to transfer a patient to an ED bed.^2^ As a result, ambulances are prevented from returning to service to be available for the next emergency medical services (EMS) call. There have been attempts nationwide to alleviate these burdens on the healthcare system.^1^,^3^,^4^ Although multiple studies have concluded that hospital-wide operational changes have a greater impact on ED crowding than attempts to divert ambulances to less busy EDs, the literature lacks consistent methods of defining and measuring intervals to determine the efficacy of policy changes on ED crowding and ambulance offload delay.^1^,^3^,^4^

The transport of emergency patients to EDs that are already overwhelmed contributes to a delay in patient offload and care transition.^5^ The Baltimore City Fire Department (BCFD) is an urban EMS jurisdiction in Baltimore City, Maryland, that responds to over 150,000 requests for EMS services per year. ED crowding and ambulance offload delay are issues within this jurisdiction that have received the attention of the local government. The BCFD initiated the medical duty officer (MDO) position in an effort to reduce ambulance turnaround time through a more proactive and informed routing of ambulances. In addition to informing decisions about a particular transport destination, the MDO was authorized to actively communicate with hospital ED representatives in an effort to more evenly distribute the transport workload throughout the jurisdiction’s hospitals, especially during times of significant ED crowding. The MDO position was a jurisdictional attempt to affect one component of the larger issue of ambulance demand and ED crowding.

It was hypothesized that any real-time intervention suggested by the MDO would be manifested in a decrease in the EMS at-hospital time, in-hospital recorded alert and diversion time and in the need for non-EMS units to wait on scene until a transporting unit was available.

## METHODS

### Jurisdiction

The studied jurisdiction is a combined fire-based EMS system serving an urban population of roughly 622,000.^6^ EMS operations are carried out with 24 full-time advanced life support ambulances and four additional ambulances during peak hours. The jurisdiction responds to roughly 150,000 calls for service per year. EMS units transport to 11 area hospitals, which include two high level trauma centers and two Level II trauma centers, as well as specialty referral centers for eye trauma, hand/upper extremity trauma, hyperbaric medicine, neurotrauma, pediatric trauma, and burns.^7^

### Medical Duty Officer

The MDO program was implemented on November 11, 2013, to proactively manage the city’s EMS resources. A veteran EMS paramedic with several years of experience was assigned to the position of the MDO. This senior EMS officer was placed in the fire communications bureau from the hours of 9 a.m. to 9 p.m., seven days a week. The MDO staffing interval corresponded with times of increased requests for EMS. In addition to monitoring EMS unit availability, the EMS officer had the operational authority to suggest alternative hospital destinations in the event that one receiving facility was experiencing delays. The MDO monitored the computer-aided dispatch (CAD) system in real time and provided feedback to responding medic units about the relative availability of ED resources. Similarly, hospital EDs that experienced a temporary surge of activity could call into the MDO and request an “internal bypass.” The internal bypass, once authorized, would temporarily reroute ambulances away from that ED.

### Data management

All aspects of this study were completed with approval of the University of Maryland School of Medicine Institutional Review Board. This study uses data collected from the BCFD CAD system. The intervals analyzed using the CAD data include the following: response time (from dispatch to arrival on scene) and at-hospital times (from arrival at the hospital to back in service). The total number of incidents and hospital transports were also pulled from the BCFD CAD system. The data set was cleaned for all non-Baltimore City transport units. We only included at-hospital times if they could be matched with a valid hospital CAD designation.

The Maryland Institute for Emergency Medical Services Systems (MIEMSS) maintains data on hospital bypass and diversion. We downloaded information about MIEMSS alerts such as yellow alert, red alert and re-route directly from the public MIEMSS Region 3 County/Hospital Alert Tracking System (CHATS) website.^8^ “Red alert” is used when a hospital has no available electrocardiogram-monitored beds. Hospitals request “yellow alert” status when the ED is subjectively overwhelmed. Yellow alert temporarily diverts all priority 3, or non-emergent, ambulance patients away from the ED.^9^ Finally, “re-route” occurs when an EMS jurisdiction places a hospital on complete bypass due to unacceptable delays in care transfer. “Re-route” is unique in that it is an alert triggered by the EMS jurisdiction.

Like urban EMS jurisdictions, the BCFD dispatches fire suppression apparatus (engine and truck companies) to certain time-sensitive medical emergencies such as cardiac arrests and shootings. Fire response is also requested in the event of a protracted delay in transport unit arrival. The BCFD classifies this type of a response as a “medic stand-by.” We therefore analyzed this interval as a surrogate marker for EMS system workload. The total time that suppression units remained on scene until ambulance arrival is recorded as “suppression wait time.”

A summary of the intervals collected and analyzed can be found in Table 1.

### Data analysis

The data analysis used a pre/post intervention design to examine the MDO impact on the BCFD EMS system. The MDO program was implemented on November 11, 2013. We defined the pre-intervention period as August 2013 - October 2013. The washout period included the entire month of November 2013, during which the MDO was implemented as a trial. The post-intervention was defined as December 2013 – February 2014. We also compared the post-intervention period to the same time period from the previous year, December 2012 – February 2013, which is referred to as the “seasonal match control.” The analysis focused primarily on the average at-hospital interval of BCFD EMS units. We examined the distribution of the at-hospital, response and suppression unit wait-time intervals for normality to determine if parametric methods were justified. We also created regression models to control for potential confounding variables captured in the dispatch data.

## RESULTS

A total of 15,567 EMS calls occurred during the pre-intervention period. The total number of EMS calls during the post-intervention period was 13,921. There was a total of 14,699 EMS calls during the “seasonal match control” period. At-hospital and response times were normally distributed during our study period.

### At-Hospital times

**Figure 1:**
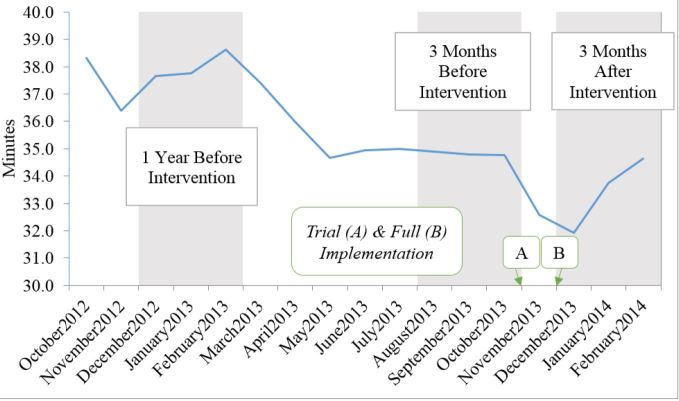
Mean at-hospital times, (time spent by ambulances at hospitals), October 2012–February 2014.

The average at-hospital time in pre-intervention period was 34.82 minutes (95% confidence interval [CI 34.57–35.08]) compared to 33.47 minutes (95% CI [33.23–33.70]) in the post-intervention period, representing a 1.35-minute (95% CI [1.01–1.70], p<0.0001) decrease. The average at-hospital time from the previous year December 2012 – February 2013 was 38.00 minutes (95% CI [37.69–38.31]), representing a 4.53-minute (95% CI [4.14–4.92], p<0.0001) decrease in the post-intervention period. The average at-hospital time while the MDO was off-duty during the post-intervention period was 31.99 (95% CI [31.74–32.24]) minutes or 1.48 (95% CI [1.14–1.82], p<0.0001) minutes shorter than on-duty times during the post-intervention period. The intervention decreased at-hospital intervals by 1.99 (95% CI [1.56–2.41], p<0.0001) minutes even after controlling for call volume and month of year (Table 2, Figure 1).

### Response times

The average response time in the pre-intervention period was 10.15 minutes (95% CI [10.05–10.26]) compared to 10.04 minutes (95% CI [9.93–10.15]) in the post-intervention period, representing a 0.11 minute decrease (95% CI [−0.04–0.27], p=0.147). The average response time from December 2012 – February 2013 was 10.82 minutes (95% CI [10.71–10.94]), representing a 0.78 minute (95% CI [0.62–0.94], p<0.0001) decrease compared to the post-intervention period. The average response time while the MDO was off duty during the intervention period was 9.36 (95% CI [9.26–9.46]) minutes, or 0.68 minutes (95% CI [0.53–0.82], p<0.0001) less than response times during post-intervention on-duty times (Table 2).

### Suppression Wait Time

The median suppression vehicle wait time in the pre-intervention period was 2.35 minutes (interquartile range [IQR 0.00–6.12]) compared to 2.05 minutes (IQR [0.00–5.68]) in the post-intervention period, representing a 0.30 minute decrease (p<0.001). The median suppression wait time from December 2012 – February 2013 was 3.28 minutes (IQR [0.11–7.99]), representing a 0.93 minute (p<0.001) decrease compared to the post-intervention period. The median suppression wait time while the MDO was off duty during the post-intervention period was 1.25 minutes (IQR [0.00–4.42]) or 0.8 minutes less than on-duty times (p<0.001, Table 2).

### Hospital alert times

**Figure 2:**
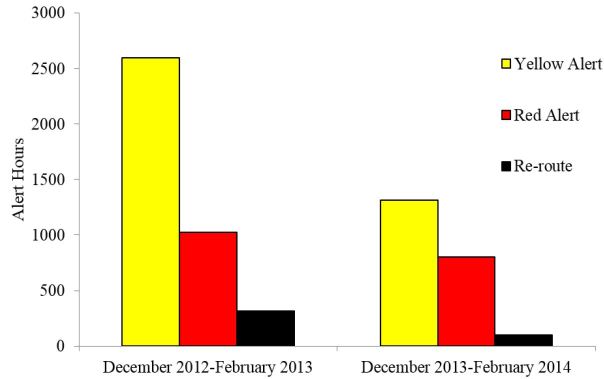
Number of total alert hours for 11 area hospitals.

The total systemwide hospital alert time (yellow alert, red alert, or reroute) in pre-intervention period was 3,937 hours (2,593 yellow, 1,027 red, 316 reroute) compared to 2,214 hours (1,315 yellow, 800 red, 99 reroute) in the post-intervention period, representing a 1,723 hour decrease in the total number of alert time between the three-month pre- and post-intervention periods. (Table 2, Figure 2).

## DISCUSSION

The MDO program was implemented to proactively manage the BCFD’s resources for emergency medical response, which included both EMS and fire apparatus. The study was performed in a fire-based EMS jurisdiction that embraces an advanced life support response structure for both first response and transport requests. As utilization of healthcare increases nationally, EDs find themselves crowded and under staffed.^1^ The transport of emergency patients to EDs that are already overwhelmed contributes to a delay in patient offload and care transition.^5^ We analyzed the effect of the MDO program and proactive ambulance routing at reducing ambulance at-hospital time, MIEMSS-recorded alert and diversion time, and suppression wait time.

**Figure 3:**
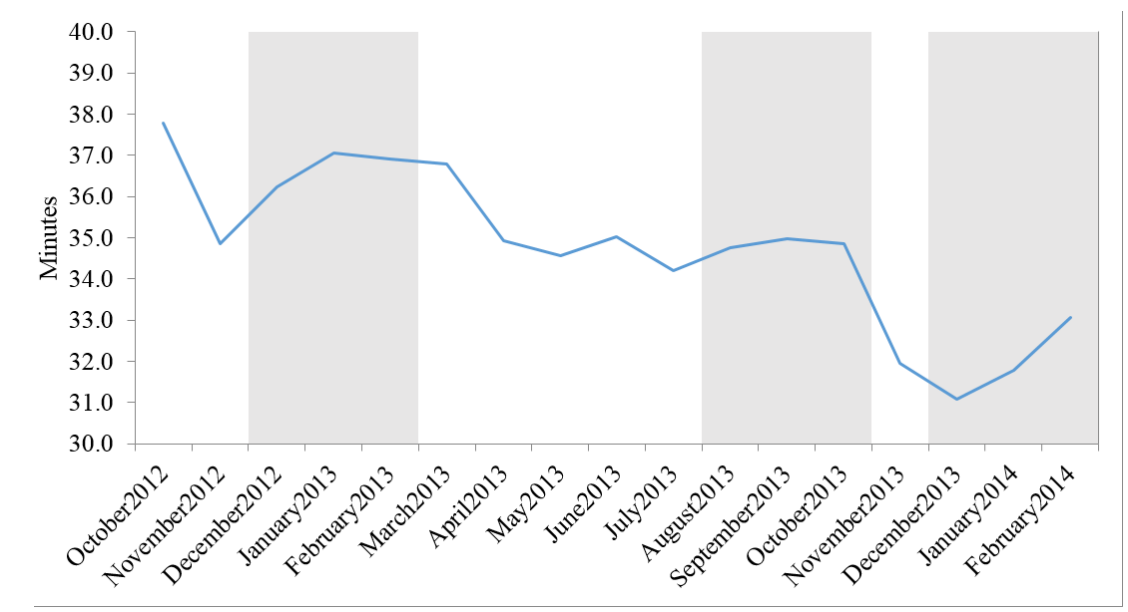
Mean at-hospital interval without medical duty officer on duty October 2012–February 2014.

**Figure 4:**
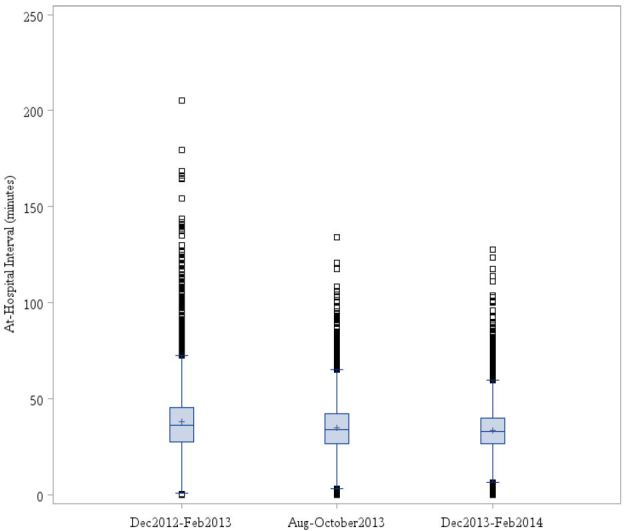
Reduction of outlying times and tighter clustering of at-hospital intervals in the time periods studied: pre-intervention period (August–October 2013), post-intervention (December 2013–February 2014) and “seasonal match control” (December 2012–February 2013).

The Spaite model of EMS time intervals defines ambulance “out-of-service interval” as the time from an EMS unit receiving the alarm to the time the unit has transferred patient care to ED staff and is again available for service.^10^ Cooney et al. expanded the concept by defining ambulance “turnaround interval” as the time from unit arrival at the hospital to the time the unit leaves the hospital, which is how we define ambulance “at-hospital time.”^4^ Therefore, factors that affect ambulance at-hospital time also impact the ambulance out-of-service interval. Various factors can contribute to extended at-hospital intervals including lack of ED beds and lack of medical personnel to receive patient information.^2^,^4^ Delaying ambulances at the hospital means they are not available for the next 911 call and therefore more ambulances are required in a given time period to achieve the same level of availability.^2^,^11^ The analysis of ambulance at-hospital time revealed a statistically significant reduction in the three-month post-intervention period compared to the three-month pre-intervention period and seasonal match control. A further analysis showed a decrease in at-hospital time post intervention while the MDO was off duty. This brought into question the impact of systemwide policy changes that were occurring concurrently versus the direct impact of the MDO on at-hospital time (Figure 3). Of particular interest, the implementation of the MDO was temporarily linked to a reduction in outlying at-hospital intervals (Figure 4). Prior to the MDO program, it was not uncommon to have units at the hospital in excess of 120 minutes. The MDO program resulted in a tighter clustering of at-hospital intervals and a modest improvement in ambulance turnaround time. Theoretically, shorter at-hospital intervals means improved EMS efficiency and public safety since ambulances are available to be dispatched on the next EMS call.^4^,^11^

Another measure of the impact on EMS and fire services is suppression wait time. The interval looks at the length of time a non-transporting fire apparatus was on the scene of an EMS call while waiting for the arrival of an apparatus with the ability to transport the patient, if necessary. Reduction of suppression wait time may theoretically increase the availability of fire apparatus for non-EMS related calls. It also represents the availability of EMS apparatus for EMS calls. If resources were appropriately available, it can be deduced that there would not be a need for suppression units to wait on-scene for the arrival of transporting units. There was a statistically significant reduction in suppression wait time post-intervention compared to both the three-month pre-intervention period and seasonal match control period. In addition, an analysis of the post-intervention MDO off-duty times actually showed a shortened suppression wait time than post-intervention MDO on-duty times.

To analyze the effect of the MDO upon the larger EMS system, we looked at the total number of hospital bypass and diversion hours. In the early 1990s, ambulance diversion programs were initiated in busy urban systems across the nation to begin to address the growing issue of ED crowding.^4^ In the more recent years, ambulance diversion has been shown to have little effect on ED crowding since ED crowding has been attributed to bigger healthcare and hospital-wide issues.^1^,^4^,^3^ However, diversion hours and alert times are still an important factor to analyze as markers of system efficiency since hospital-based policy changes have been shown to reduce ED crowding and therefore decrease ambulance diversion.^4^ There is also a need for common variables, such as alert times, to have the ability to compare the efficacy of interventions and policy changes being made nationwide.^1^ After implementation of the MDO program, there was just over a 1,000-hour reduction in total alert times from the three-month pre-intervention period to three-month post-intervention period. Unfortunately, we are limited in our ability to further investigate the data. In addition, we are unable to compare the alert hours when the MDO was on duty to when the MDO was off duty in the post-intervention period. Statewide alert data are not reported on an hourly basis and therefore could not be uniformly adjusted for the MDO time intervals. In the future, hospital alert times could be an effective means for determining the effect of interventions on the hospital system if we are able to further navigate and analyze the data.

There is a paucity of literature that addresses the topic of proactive dispatch as it relates to reduction in at-hospital times or related intervals. Ambulances represent a valuable resource to the community and extended at-hospital times have the potential to reduce ambulance availability.^2^,^5^,^4^,^11^ Accordingly, it is imperative for EMS jurisdictions to consider strategies targeted to maintain their capacity for emergency response. The MDO program’s findings are similarly relevant to EMS jurisdictions that deal with diversion and transport to multiple hospitals. Though MIEMSS maintains a statewide alert-reporting database, hospitals may tailor diversion criteria to fit their operational constrains. Despite recommendations against excessive ambulance offload times, the practice of timely patient offloading is unevenly enforced at area hospitals in our jurisdiction. The MDO program therefore represents one urban EMS jurisdiction’s attempt to maintain real-time situational awareness and attempt to adopt a more proactive ambulance deployment strategy. Reductions in at-hospital, response times, suppression wait time and hospital alert times were modest, at best, and most likely reflect a temporally associated and jurisdiction-wide hospital collaboration initiative. Future iterations of this paper would apply the MDO program to other EMS jurisdictions and see if it has similar effects on the measured intervals without other factors confounding the results. If so, it would support the clinical utility of this program. In addition, the cost effectiveness of the MDO program was not considered prior to implementation. Future iterations would add cost analysis to the program and determine if the fiscal impact of decreasing suppression wait time and increasing ambulance availability increases profit, even with the increased cost of staffing the senior officers of the MDO program. At this time there is no study that shows reducing ED crowding saves money.

Finally, it is difficult to argue that small reductions in response times equate to clinically meaningful results. Response time has been deemphasized as a measure of clinical efficacy, and it is cited in this study solely as a marker of EMS unit availability. In our EMS jurisdiction, the at-hospital interval represents the longest amount of time that a transport unit is effectively taken out of service. When viewed from a systemwide perspective, small improvements in the at-hospital interval can translate into improved ambulance availability. Even in the absence of clinically significant improvements, the authors believe there is value in tracking intervals and engaging hospitals in an effort to reduce at-hospital times. The MDO program has resulted in more regular communication between the EMS jurisdiction and area EDs, and the effects of inter-agency collaboration extend well beyond improvements in established time intervals. At a minimum, the results and limitations associated with this particular investigation reiterate the need for ongoing communication and consistent data collection between EMS units and the hospitals they serve.

The MDO project allowed for a uniform reporting of variables and time intervals. A consistent vocabulary is absolutely essential for ongoing and meaningful dialogue. Prior to inception, each hospital had a different way of scrutinizing the “at-hospital” interval. Fire department administrators resorted to manually charting the interval between ambulance arrival in the ED and eventual unit availability. The MDO project encouraged use of the intervals described in the Spaite model to ensure that stakeholders were measuring, analyzing, and understanding specific and uniform times.^10^ The need for consistency in measurement cannot be overstated.

## LIMITATIONS

Many limitations exist in the availability and recording of data used in this paper. The tracked intervals used begin and end when the provider, Fire Communications, or the EMS officer manually changes the unit’s status in the CAD system. The lack of automated time stamps allows for inconsistencies in reporting. Furthermore, it is entirely possible that crews forgot to communicate the exact time of their arrival to fire communications and therefore under or overestimate actual intervals. The MIEMSS Alert System has no means of tracking when the MDO made destination changes or accepted internal bypass requests. Our jurisdiction also encountered challenges with consistent implementation of automated Wi-Fi-enabled reporting. The physical structure of some area hospitals, for a variety of reasons outside the BCFD’s control, prevented the transmission of wireless time-stamp data. In addition, there is no research that directly links response times to improved health outcomes, or research that relates the quantity of alert hours to EMS efficiency.

BCFD units transport to 11 hospitals within their catchment area. Area hospitals did not report EMS time metrics in a uniform manner, nor was information shared with the EMS jurisdiction at the time of the study’s inception. Therefore, the average at-hospital interval represents the only metric that is collected, reported, and analyzed at the jurisdictional level. To address the variability intrinsic to the reporting of “average” time intervals, the at-hospital interval was examined pre- and post-intervention as well as controlled for the volume of responses.

The study was not resourced to address the ancillary benefits of positioning an experienced EMS officer in fire communications. Units frequently used MDO for issues unrelated to hospital availability. Experienced MDOs reported fielding requests for “advice” on appropriate destination when medics in the field were faced with complicated questions about patient destination. Future studies might focus on the potential reduction in questions to medical control physicians or use a survey-based response system to more precisely characterize benefits and perceived MDO utility.

Finally, the city’s MDO program was implemented during a time when there was increased scrutiny on EMS at-hospital times and multiple efforts had been made to outreach to city hospitals. A city-wide hospital collaboration group was convened to re-address the problem of at-hospital intervals and improve offload times. As a result, the MDO program is only one factor contributing to changes in the EMS system during the time frame studied in this paper.

## CONCLUSION

A proactive deployment of a designated medical duty officer is associated with a small, contemporaneous reduction in average at-hospital times within an urban EMS jurisdiction. The clinical utility of the findings in this paper is debatable. However, this project highlights the importance of better communication between EMS systems and area hospitals as well as uniform reporting of variables. More research is required to determine the precise influence of a proactive EMS officer presence in the fire communications center on relevant EMS time intervals and hospital crowding.

## Figures and Tables

**Table 1:** Description of hospital bypass and diversion intervals collected and analyzed for this study.

Interval	Description	
Response time	Dispatch to arrival on scene	
At-hospital time	Arrival at hospital to back in service	
Suppression wait time	Time suppression units remained on scene until ambulance arrival	
Hospital alert time	Red alert	No ECG-monitored beds available
	Yellow alert	Diverts non-emergency ambulance patients away from ED
	Re-route	Complete bypass due to delays in care transfer from ambulance to ED staff

*ECG,* electrocardiogram; *ED,* emergency department

**Table 2:** Response metric pre-intervention averages compared to post-intervention averages after a medical duty officer was hired to act as liaison between the Baltimore City Fire Department and area emergency departments.

Response Metric	Seasonal Match Control	Pre-Intervention	Post-Intervention	Off-Duty Post-Intervention
Time Frame	Dec 2012–Feb 2013	Aug 2013–Oct 2013	Dec 2013–Feb 2014	Dec 2013–Feb 2014
Mean At-Hospital times (min)	38.00**	34.82	33.47*	31.99**
Mean Response times (min)	10.82*	10.15	10.04	9.36**
Hospital Alert times—Yellow (hrs, total)	***	2593.96	1315.09	***
Hospital Alert times—Red (hrs, total)	***	1027.19	800.57	***
Hospital Alert times—Reroute (hrs, total)	***	316.73	99.07	***
Median Suppression Unit Standby times (min)	3.28**	2.35	2.05*	1.25**

* Statistically significant difference from pre-intervention period (p<0.05, 95% CI)

** Statistically significant difference from on-duty post-intervention time (p<0.05, 95% CI)

*** Data unavailable
